# Right retrocaval ureter type 2 with left atrophied kidney: A rare case report

**DOI:** 10.1016/j.eucr.2025.102936

**Published:** 2025-01-11

**Authors:** Mustafa I. Al-Shalah, Zaid F. Altawallbeh, Rashed Yousef Al Sharqi, Mohammad kh Alzawahreh, Raed Bassam Abulawi, Mohab Alsaid Saad, Yousif Ahmad Hanafi, Laith Alnajada, Ala' Mohammad Yaser Alfreahat

**Affiliations:** a Department of Urology, Al Basheer Hospital, Amman, Jordan; b Al Nadeem Hospital, Madaba, Jordan; c Department of Urology, Ministry of Health, Amman, Jordan; d Faculty of Medicine, Al-Azhar University, Cairo, Egypt; e Al-Azhar University Faculty of Medicine, Cairo, 11884, Egypt; f University of Jordan Hospital, Jordan; g Al Basheer Hospital, Amman, Jordan

**Keywords:** Retrocaval ureter, Type 2 retrocaval ureter, Flank pain, Pyelo-pyelostomy

## Abstract

**Case:**

A 22-year-old male smoker presented with intermittent right flank pain lasting over a year. He had a history of atrophied left kidney and gout. Physical exam revealed mild right renal angle tenderness.

**Outcome:**

Initial imaging, pointed to a diagnosis of ureteropelvic junction stenosis. During surgery, a type two retrocaval ureter was discovered. Transposition pyelo-pyelostomy was performed to repair the ureter, and a double-J stent was inserted.

**Conclusion:**

This case highlights the challenge of accurately diagnosing retrocaval ureter, especially type, based on initial radiological images.

## 1 Introduction

Retrocaval or circumcaval ureter, also known as pre-ureteral vena cava, is a rare congenital anomaly that almost always affects the right side and has a global incidence rate of 0.06–0.17 %.^1^ Men are three times more likely than women to have it.^2^ The infrarenal segment typically develops from the supracardinal vein; however, in the retrocaval ureter, the right posterior cardinal vein—located anteriorly and laterally to the ureter—develops the infrarenal IVC. Consequently, the proximal ureter becomes trapped behind the IVC. Often, this is asymptomatic; however, patients with retrocaval ureter may experience dull aches or intermittent flank or abdominal pain in their third and fourth decades of life due to ureteric obstruction and associated hydronephrosis.^3^ Ureter kinking or compression against the psoas muscle could be the cause of the hydronephrosis.^4^ Urinary tract infections (UTIs) can also occur in patients with retrocaval ureters. On intravenous urography (IVU), the ureter projects over or medial to the lumbar pedicles, taking an irregular course.^5^ In these cases, the ureter is surgically situated anterior to the IVC.

## 2 Case presentation

A 22-year-old single, smoker, male, presented to our specialized urology clinic, department of Urology at Al-Nadeem hospital in Jordan with intermittent right flank pain for more than 1 year. The pain was colicky in nature, intermittent, increased with intakes of fluids, and decreased by analgesia. There was no history of fever or vomiting. His past medical history is significant for atrophied left kidney, gout since 2016, and his father had PUJ stenosis. General physical examination showed mild right renal angle tenderness. Other systems were normal. The results of the laboratory tests are shown in Table 1.

**Table 1:** Laboratory investigation results.

Blood	
Red blood cells	5.33
Hemoglobin	16.6
Wight blood cells (WBC)	9.47
MCHC	36.6
MCH	31.1
MCV	85.2
PCV	45.4
Platelets	158
RDW-SD	36.1
RDW-CV	11.7

Serum		
Creatinine	74	umol/L
Glucose	4.48	mmol/L
Urea	3.3	mmol/L
Chloride	100.8	mmol/L
Potassium	4.37	mmol/L
Sodium	138	mmol/L

**Urine**	
Glucose	NULL
protein	NULL
WBC	1–3
RBC	0–2
Bacteria	NULL
Ketones	NULL
Bilirubin	NULL
Urobilinogen	NULL
Color	Yellow

**Plasma**		
PT	13.3	Seconds
PTT	29.6	Seconds
INR	1.02	

**Fig. 1:**
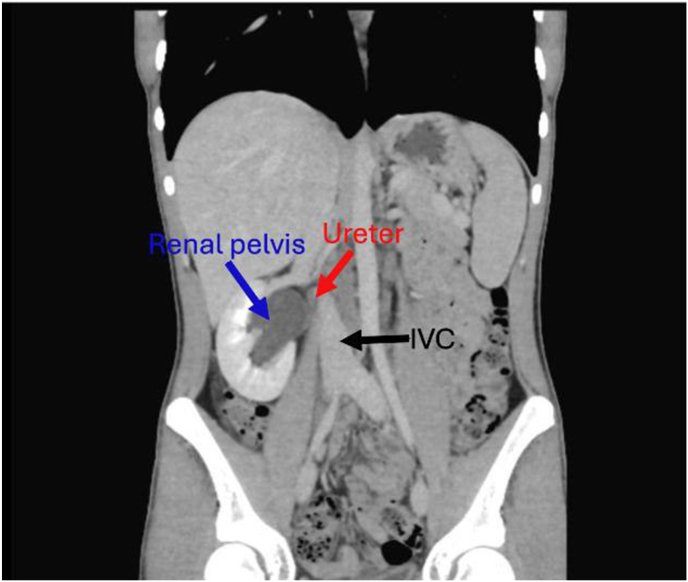
**Renal CT scan with contrast.**

**Fig. 2:**
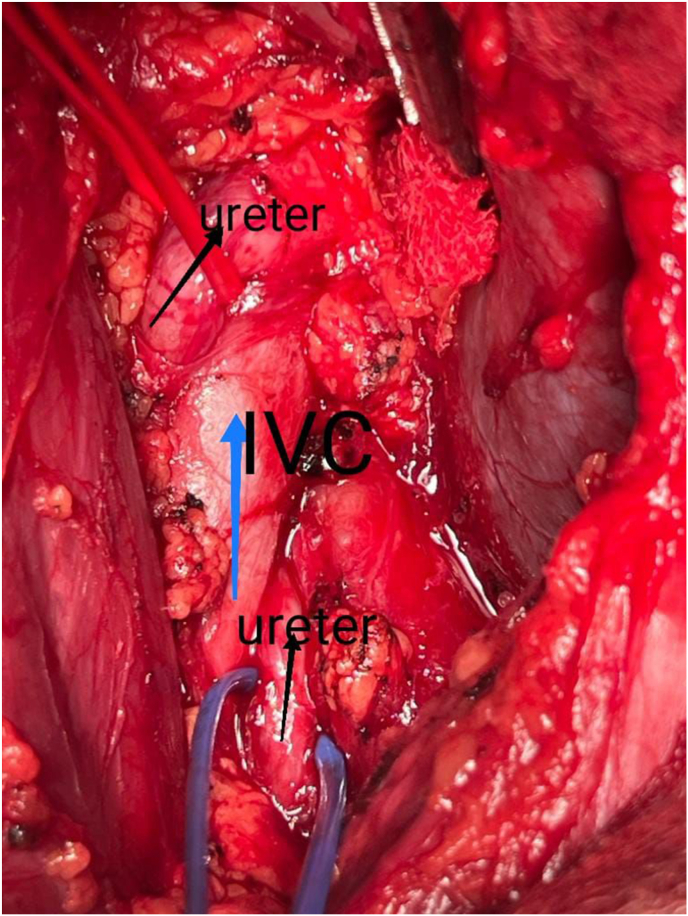
**type 2 Retrocaval ureter discovered in the operating room.**

**Fig. 3:**
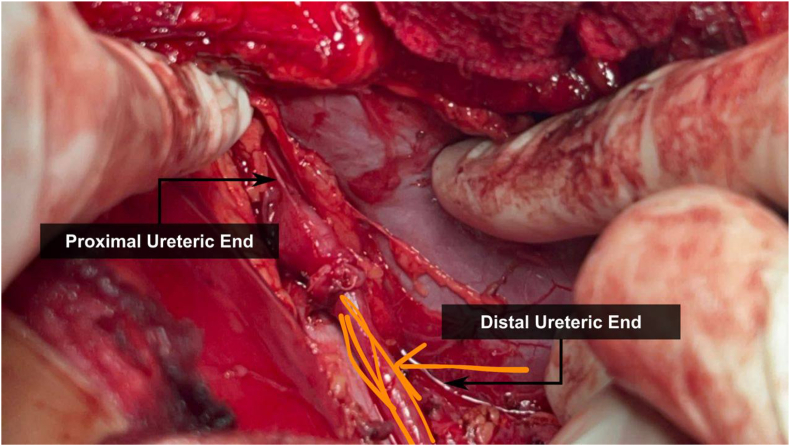
**Transposition pyelo-pyelostomy for repair of reterocaval ureter.**

An abdominal ultrasonography revealed moderate right renal hydronephrosis with atrophied left kidney. A routine X-ray kidney-ureter-bladder (KUB) was normal. Renal CT scan with contrast revealed right PUJ stenosis, and small atrophied poorly functioning left kidney as shown in Fig. 1. Depending on these results, the patient was diagnosed with Ureteropelvic stenosis, for which he went for pyeloplasty. After taking the patient's consent, we approach retroperitoneal space through intercostal flank incision. Incidentally, we found that the proximal ureter was passing behind the Inferior Vena Cava (IVC) as shown in Fig. 2. Then transposition pyelo-pyelostomy for repair was done as shown in Fig. 3.

**Fig. 4:**
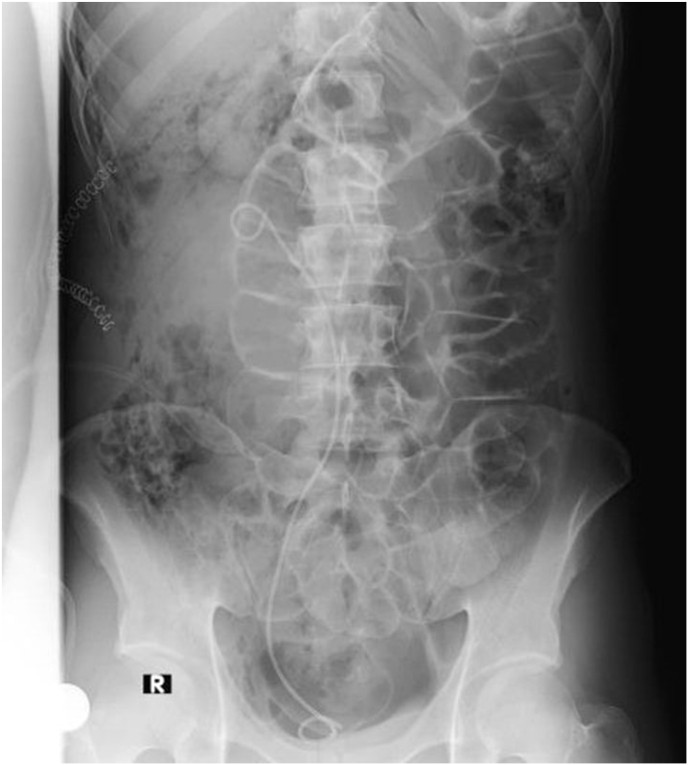
**Post-operative renal CT scan showing inserted double J.**

We inserted a double-j stent, and the patient was discharged three days later after the operation as shown in Fig. 4. We prescribed the patient Paracetamol for pain management. After a month, there were no complications, and we removed the double-j stent. Three-month follow-up demonstrates complete resolution of this pathology.

## 3 Discussion

To our knowledge, this is the first reported case of retrocaval ureter type 2 in Jordan. Retrocaval ureter is a rare congenital anomaly in which the proximal part of the ureter passes medially and behind the inferior vena cava, causing it to be compressed under the pressure of the IVC. This anomaly occurs most often on the right side,^6^ although it is extremely rare, it can occur in the left side as in situs inversus or IVC duplication.^7^

Most patients are clinically silent; however, few patients might present with nonspecific symptoms that mimic ureteropelvic junction obstruction (UPJO) or urinary stone disease^1^^,^^8^

There are two types of retrocaval ureter; type 1 in which the ureter cross behind the IVC at the level of third lumber vertebra and it is associated with a higher incidence of marked hydronephrosis. Characteristic radiological appearance of this type is the “Fish Hook” deformity at the level of ureter obstruction.^9^

In this case study we present the less common type of retrocaval ureter (type 2), where the ureter cross behind the IVC at the level of renal pelvis. The patient was first diagnosed with a pelvic ureteric junction obstruction depending on the doctor's initial impression upon clinical examination, abdominal ultrasonography, KUB x-ray and renal CT scan with contrast. This might refer to the decreased accuracy of these radiologic images to diagnose a case with retrocaval ureter, especially if it was type 2.^10^

Surgery is considered the right definitive treatment for symptomatic patients, especially those with recurrent flank pain and moderate to severe hydronephrosis. The open surgery approach is used in most cases; the procedure involves identification and dissection of the ureter, dilated pelvis and IVC. The dilated renal pelvis is then transected. The proximal ureter is transposed anterior to IVC together with the distal ureter, then pyeloplasty is performed in a tension free, watertight manner over internal ureteral stent however, the recent studies prefer laparoscopic approach over open surgery due to several benefits, including shorter hospitalization, better outcomes, and fewer complications.^7^

In this study we did the open surgery method. After putting the patient in the right flank position and under general anesthesia, retrocaval ureter was diagnosed and the obstruction was found at the level of the renal pelvis. Transposition pyelo-pyelostomy for repair of reterocaval ureter was done.

Finally we inserted double-J Stent. Follow up of the patient had no insightful events and the case was completely resolved.

## CRediT authorship contribution statement

**Mustafa I. Al-Shalah:** Conceptualization, Investigation, Project administration. **Zaid F. Altawallbeh:** Conceptualization, Investigation, Project administration, Writing – review & editing. **Rashed Yousef Al Sharqi:** Formal analysis, Resources, Supervision. **Mohammad kh Alzawahreh:** Conceptualization, Validation, Visualization. **Raed Bassam Abulawi:** Investigation, Project administration. **Mohab Alsaid Saad:** Data curation, Formal analysis, Methodology, Validation, Writing – original draft, Writing – review & editing. **Yousif Ahmad Hanafi:** Data curation, Formal analysis, Writing – original draft, Writing – review & editing. **Laith Alnajada:** Validation, Visualization. **Ala' Mohammad Yaser Alfreahat:** Validation, Visualization.

## Declaration of competing interest

None Declared.
