# Supplementary material for: Right retrocaval ureter type 2 with left atrophied kidney: A rare case report
Source: Urol Case Rep. 2025 Jan 11;59:102936. doi: 10.1016/j.eucr.2025.102936 (PMC11782891; doi:10.1016/j.eucr.2025.102936)

**Figure 1: Pre-operative Renal CT with contrast**


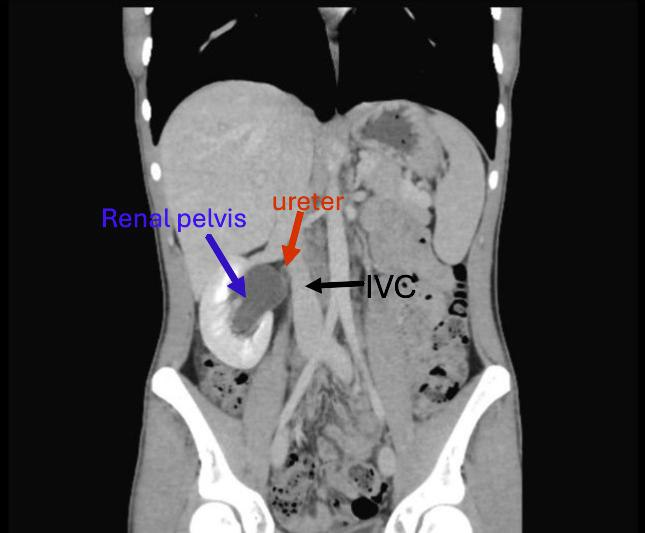


**Figure 2: Pre-operative Renal CT with contrast**


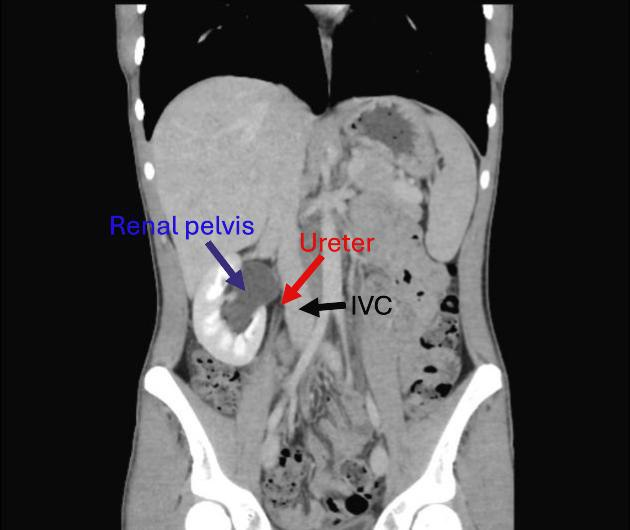


**Figure 3: Retrocaval ureter in the operating room**


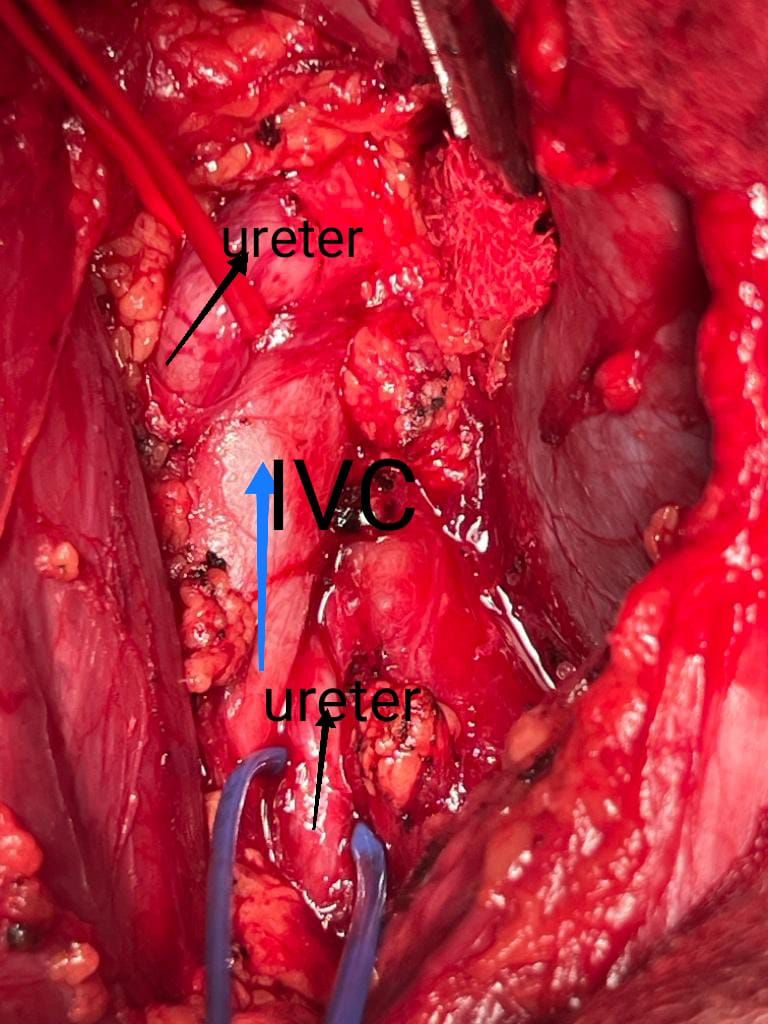


**Figure 4: Transposition pyelo-pyelostomy for repair of reterocaval ureter**


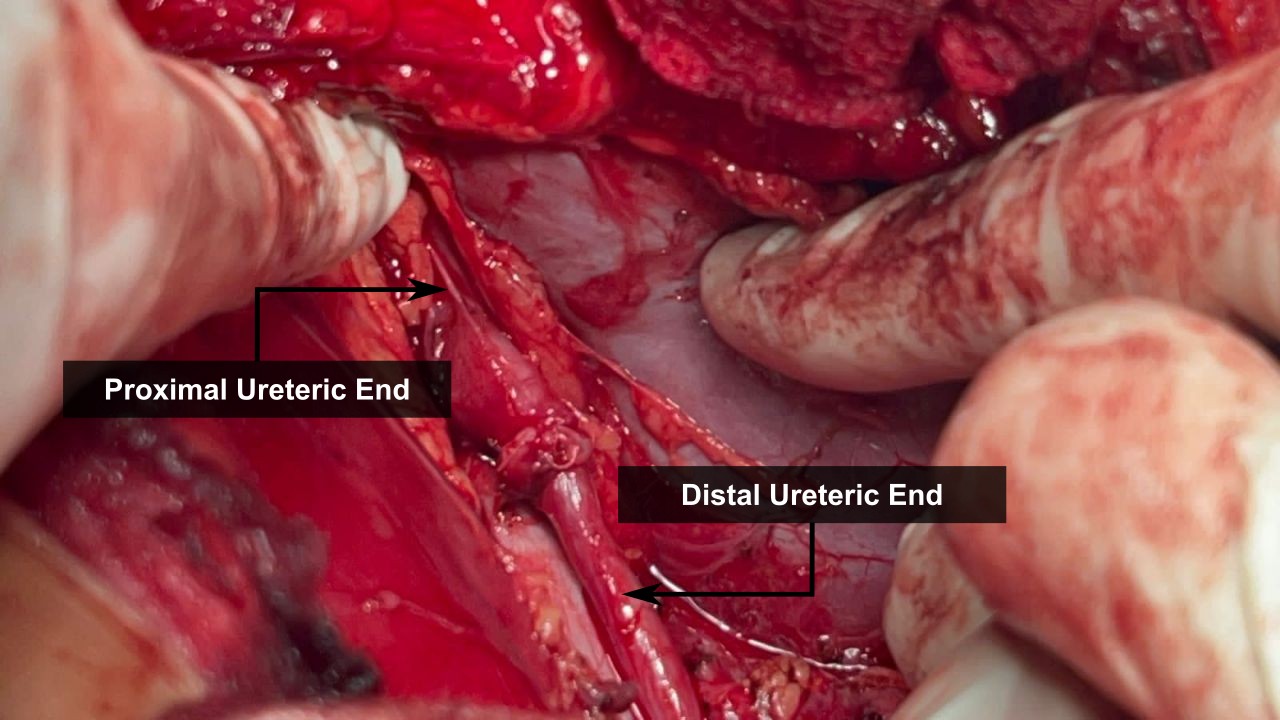


**Figure 5: Post-operative renal CT scan showing double J**


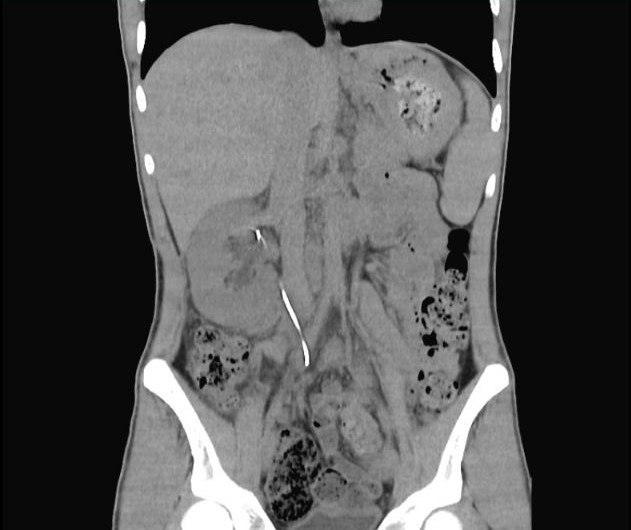


**Figure 6: Post-operative X-ray Showing double J**


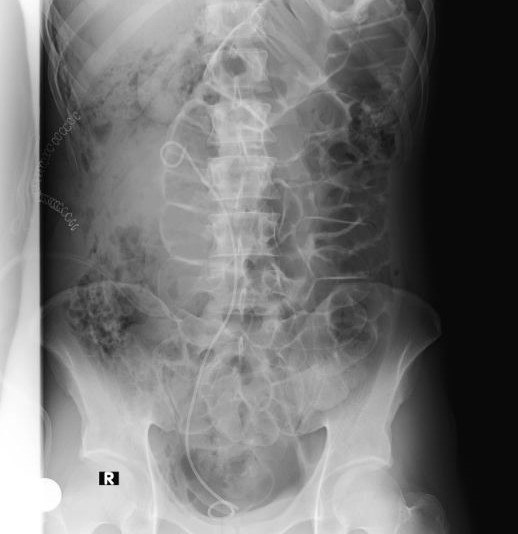

Supplement: Multimedia component 1 [file mmc1.docx]
